# The Role of Serum Calcium Levels in Pediatric Dyslipidemia: Are There Any?

**DOI:** 10.3389/fped.2021.712160

**Published:** 2021-08-09

**Authors:** Yaguang Peng, Lixin Hu, Xiaolu Nie, Siyu Cai, Ruohua Yan, Yali Liu, Yanying Cai, Wenqi Song, Xiaoxia Peng

**Affiliations:** ^1^Center for Clinical Epidemiology and Evidence-Based Medicine, National Center for Children's Health, Beijing Children's Hospital, Capital Medical University, Beijing, China; ^2^Clinical Laboratory Center, National Center for Children's Health, Beijing Children's Hospital, Capital Medical University, Beijing, China

**Keywords:** calcium, dyslipidemia, pediatric, obesity, albumin-corrected calcium

## Abstract

**Background:** No previous study explored the association between serum calcium levels and dyslipidemia in children. This study aimed to explore this relationship in children, based on a multicenter cross-sectional study population in China.

**Methods:** Cross-sectional data was derived from the Pediatric Reference Intervals in China (PRINCE) study conducted between 2017 and 2018 involving 5,252 males and 5,427 females with a mean age of 10.0 ± 4.6 years. Multivariable logistic regression models were applied to calculate odds ratios (ORs), with 95% confidence intervals (CIs), for dyslipidemia of each serum calcium level and albumin-corrected calcium levels, which were sorted into quartiles. The restricted cubic spline model was fitted for the dose-response analysis. An L-shaped dose-response relation between calcium levels and the probability of dyslipidemia was found after the adjustment for multiple potential confounding factors, *p* for non-linear < 0.001.

**Results:** Using the middle category of calcium level as the reference, multivariable-adjusted ORs and 95% CIs of the lowest and the highest quartile categories were 0.96 (0.82–1.12) and 1.29 (1.12–1.48), respectively, for total serum calcium levels and 1.06 (0.91–1.23) and 1.39 (1.21–1.60) for albumin-corrected calcium levels.

**Conclusions:** Individuals with higher levels of serum calcium were associated with increased risk of dyslipidemia in a sample of a healthy Chinese pediatric population. The association between serum calcium levels and dyslipidemia needs to be examined prospectively in future studies.

## Introduction

Calcium plays an important role in intracellular and extracellular signal transductions (1, 2) and muscle contractions (3), as well as other biological functions (4). Serum calcium level is mainly regulated by the balance of hormones (e.g., calcitonin and parathyroid hormone) and can be influenced by various factors such as diet, vitamin D levels, and daily physical activity (5–8). Recently, some epidemiological studies indicated that the serum calcium level was associated with cardiovascular diseases, including hypertension, diabetes, and insulin resistance in adult populations (9–12). Exposure to risk factors in childhood, such as abnormal glucose and lipid metabolism and obesity, increases the risk of cardiovascular metabolic disease in adulthood and has a persistent effect (13–16). In the context of the COVID-19 pandemic, the risk factors of cardiovascular diseases in children and adolescents have attracted more attention due to changes in lifestyle factors including dietary nutrition, physical activity, sleep, sedentary, etc. (17, 18). The current situation of maternal and child healthcare in China were improved greatly, and the potential excessive use of calcium supplements and the coexistence of excessive nutrition and lack of exercise among children might increase the risk of cardiovascular diseases. Few studies have focused on the association between serum calcium levels and dyslipidemia in child populations, although the calcium homeostasis is vital for children's growth and development. In this study, we hypothesized that calcium levels might be associated with lipid metabolism in child populations. For this purpose, we analyzed the biochemical analyte data from the Pediatric Reference Intervals in China (PRINCE) study population.

## Methods

### Study Population

**Figure 1:**
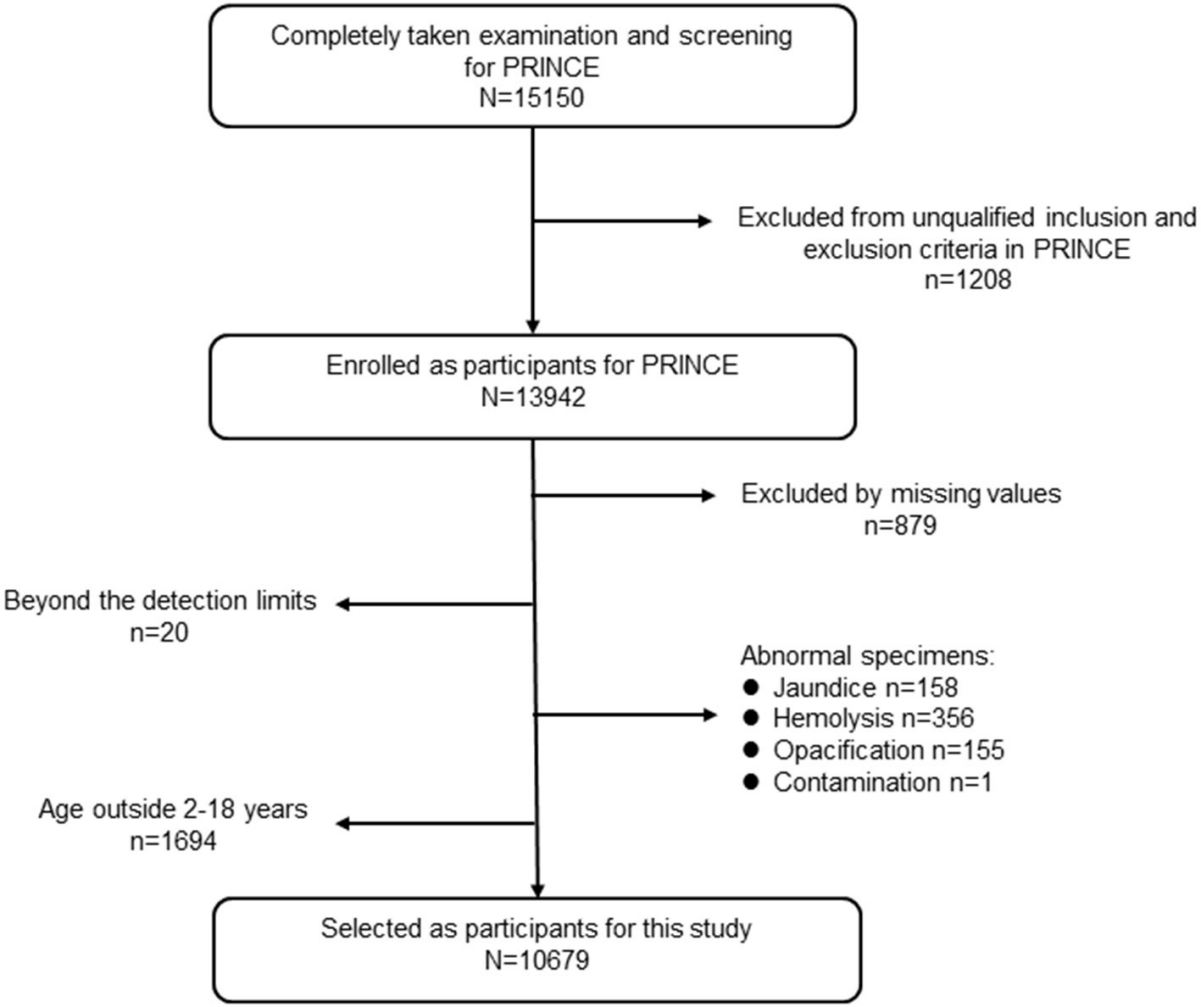
The flowchart of the study population from the PRINCE study.

Participants were derived from the PRINCE study enrolled from January 2017 to August 2018, which aimed to establish and verify pediatric reference intervals for Chinese children based on a nationwide multicenter cross-sectional study. A detailed description of the PRINCE study design and methods were published (19). Briefly, PRINCE was conducted by the Hospital Authority, National Health Commission of China. A major objective of PRINCE was to establish pediatric reference intervals (RIs) of 31 commonly used clinical laboratory parameters (18 measurands of complete blood count (CBC) and 13 biochemical analytes) based on a multicenter cross-sectional study covering 11 provinces and municipalities in China. Geographically, those centers covered the northeast (Liaoning Province), north (Beijing Municipality and Hebei Province), northwest (Shaanxi Province), middle (Henan and Hubei provinces), south (Guangdong Province), southwest (Chongqing Municipality and Sichuan Province), and east (Shanghai Municipality and Jiangsu Province) of China. The PRINCE study was composed of two phases: the establishment phase and the validation phase. The population of the establishment phase was involved in this study. In total, 15,150 participants aged 0–19 years old were screened in the PRINCE study. In this study, we only included individuals aged 2–18 years who completed the whole procedures including the epidemiology investigation, physical examination, blood sample collection, and laboratory test. After eliminating the missing values for critical variables, for instance serum calcium, serum lipid analytes, inorganic phosphate, albumin, resulted from the unqualified samples (e.g., insufficient sample volume, jaundice, hemolysis, or samples with chyle), 10,679 participants were included in the final analysis for this study. A detailed flow chart depicting participant selection is shown in Figure 1. The PRINCE study conformed to the ethical guidelines of the 1975 Declaration of Helsinki and was approved by The Institutional Review Board of Beijing Children's Hospital (IEC-C-028-A10-V.05). In addition, the protocol was approved by the institutional review boards of each collaborating centers. Informed consent was obtained from each participant's legal representative (parent or guardian) in the case of children aged 8 years or less. Additionally, it was assented by the child her/himself and consented by the legal guardian for children older than 8 years.

### Data Collection and Management

In the PRINCE study, the Internet-based electronic data capture (EDC) platform was developed and used for data collection and management. Upon the completion of laboratory testing (CBC and biochemical markers), data managers at each center exported the results from the laboratory information system to the EDC platform. The whole data collection was completed after combining the epidemiology investigation, physical examination data, detailed information for sample collection and transport, and laboratory testing results. Some raw data was recorded in paper form when using the Internet was not convenient. All the paper forms were inputted and uploaded onto the platform, and the original data was stored and reviewed by the Data and Safety Monitoring Board (DSMB) at Beijing Children's Hospital. All data corrections and revisions will be tracked and marked.

Three sections of the data from the PRINCE database were extracted for this study: basic demographic information, epidemiology investigation data, and the laboratory test data of relevant analytes. Considering the age distribution and characteristics of analytes, five age subgroups were divided for this study, as such: 2–5, 6–9, 10–12, 13–15, and 16–18 years. The age- and sex-specific BMI Z-score were calculated according to the standard methods recommended by the World Health Organization (20, 21).

### Laboratory Tests and Clinical Definitions

Participants in the PRINCE study were required to fast for at least 8 h before their blood sample collection, which occurred between 8 and 11 a.m., extracted by trained pediatric nurses using a BD Vacutainer and vacuum tube needles (Becton, Dickinson and Company, Dublin, Ireland). All blood sampling procedures to collect, handle, and store these samples were strictly in accordance with standard operating protocols (SOPs), which were reviewed and adopted by the committee of the PRINCE. After processing at each center, all the specimens were transported to the central laboratory at Beijing Children's Hospital in cold chain for analysis, which was completed within 6 months after collection, without repeated freeze-thaw cycles. The biomedical markers were measured using an automated biochemistry analyzer, Cobas C702 (Roche Diagnostics GmbH, Mannheim, Germany). The detailed procedure and quality control have been reported (19). The detection methods of biochemical analytes involved in this study were listed in Supplementary Table 1.

The diagnosis definition of dyslipidemia in children agreed upon by Chinese experts is as follows (22): (1) total cholesterol (TC) ≥5.18 mmol/L, (2) triglyceride (TG) ≥1.70 mmol/L; (3) high-density lipoprotein-cholesterol (HDL-C) ≤ 1.04 mmol/L; or (4) low-density lipoprotein-cholesterol (LDL-C) ≥3.37 mmol/L. Glycemia was defined as fasting glucose ≥7.00 mmol/L according to the American Diabetes Association (23). Albumin-corrected calcium levels (24) were calculated as follows: serum calcium (mmol/L) + 0.02 × [40-serum albumin (g/L)]. Creatinine clearance (ml/min/1.73 m^2^) was estimated by Schwartz formulas (25, 26).

### Statistical Analysis

Data was depicted as a mean and standard deviation (SD) for continuous variables and as frequency (percentages) for categorical variables. Serum calcium levels and albumin-corrected calcium levels were analyzed as categorical variables according to the 25th (P_25_) and 75th percentiles (P_75_) of the distribution, respectively. Independent *t*-tests for continuous variables and χ^2^ tests for categorical variables were used to compare differences in characteristics between the dyslipidemia group and the control group. The middle group was defined as the reference and the lower P_25_ group and the higher P_75_ group were compared with middle group in order to explore the similar trends using the logistic analysis. The odds ratios (ORs) and 95% confidence intervals (CIs) for dyslipidemia and its components of each calcium category were estimated using multivariate non-conditional logistic regression models. The status of dyslipidemia was taken as the dependent variable, and serum calcium and corrected calcium levels were taken as independent variables to fit multivariate models separately. Multivariate models 1, 2, and 3 were fitted successively considering different adjustment factors, including age, sex, BMI z-score, serum albumin levels, serum creatinine levels, alanine aminotransferase, inorganic phosphate, alkaline phosphatase, and total protein. The restricted cubic spline model was used for the dose-response analysis. Using serum-corrected calcium level as the dependent variable and age, height, weight BMI, serum albumin levels, serum creatinine levels, inorganic phosphate, alkaline phosphatase, total protein, etc. as the independent variables, the quality association between serum calcium level and related anthropometric indices and biomarkers were analyzed by multiple linear regression model. A two-tailed *p* < 0.05 was considered significant. All statistics were conducted using SAS version 9.4 (SAS Institute, Cary, NC, USA) and R version 3.6.3 (R Foundation for Statistical Computing, Vienna, Austria).

## Results

The characteristics of this population are depicted in Table 1. There were a total of 10,679 participants, 5,252 males and 5,427 females, with a mean age of 10.0 ± 4.6 years. The dyslipidemia rate was 12.7% in males and 13.6% in females, respectively. The sex-specific rates of components, high TC, high TG, high LDL-C, and low HDL-C were 3.5, 3.2, 4.1, and 5.1% in males and 4.3, 4.2, 4.8, and 4.3% in females. With exception of the high LDL-C rates with *p*-value 0.053, other differences above between sexes were significant (*p*-values were 0.024, 0.008, and 0.043 for high TC, high TG, and low HDL-C). Biochemical analytes in this study indicated the age-dependent trend.

**Table 1:** Characteristics of the study population (*n* = 10,679).

	**Female**					**Male**				
	**2–5 years**	**6–9 years**	**10–12 years**	**13–15 years**	**16–18 years**	**2–5 years**	**6–9 years**	**10–12 years**	**13–15 years**	**16–18 years**
N	1,164	1,458	1,041	910	854	1,311	1,547	1,081	788	525
Age, year	4.00 ± 1.11	8.04 ± 1.13	11.47 ± 0.90	14.53 ± 0.92	17.48 ± 0.87	3.91 ± 1.10	8.02 ± 1.12	11.48 ± 0.90	14.48 ± 0.92	17.30 ± 0.85
Height, cm	102.67 ± 9.52	128.52 ± 8.90	149.28 ± 9.14	159.23 ± 6.38	160.77 ± 6.08	103.30 ± 9.40	129.41 ± 9.08	149.14 ± 10.07	167.14 ± 8.72	172.91 ± 6.54
Weight, kg	16.53 ± 3.59	27.01 ± 6.43	41.13 ± 10.06	51.98 ± 9.70	55.48 ± 8.57	17.00 ± 3.61	28.83 ± 7.96	43.80 ± 12.77	57.80 ± 13.51	64.81 ± 12.34
BMI, kg/m^2^	15.55 ± 1.66	16.18 ± 2.44	18.28 ± 3.06	20.45 ± 3.33	21.45 ± 2.89	15.81 ± 1.64	17.00 ± 3.26	19.41 ± 4.05	20.53 ± 3.84	21.67 ± 3.68
BMI z-score	0.06 ± 1.10	0.02 ± 1.16	0.06 ± 1.14	0.03 ± 1.04	0.00 ± 0.87	0.19 ± 1.19	0.41 ± 1.52	0.57 ± 1.43	0.18 ± 1.24	−0.07 ± 1.22
Total protein, g/L	69.82 ± 3.77	73.61 ± 3.83	75.17 ± 3.94	78.08 ± 4.25	77.52 ± 3.99	69.50 ± 3.61	73.25 ± 3.74	75.22 ± 3.73	77.17 ± 4.06	77.02 ± 3.87
Albumin, g/L	47.64 ± 2.27	48.87 ± 2.38	49.34 ± 2.39	50.49 ± 2.53	50.17 ± 2.35	47.47 ± 2.23	48.45 ± 2.26	49.53 ± 2.38	50.97 ± 2.66	51.34 ± 2.37
Creatinine, μmol/L	32.80 ± 5.38	42.36 ± 5.77	47.58 ± 7.45	57.13 ± 8.81	61.08 ± 8.13	33.20 ± 5.45	43.40 ± 6.20	51.43 ± 7.61	67.32 ± 12.65	79.64 ± 11.06
Alanine aminotransferase, U/L	13.46 ± 4.63	13.16 ± 5.99	12.94 ± 6.66	12.38 ± 5.85	12.76 ± 7.31	14.05 ± 7.78	14.78 ± 13.02	16.34 ± 14.59	16.36 ± 14.69	18.68 ± 15.21
Aspartate aminotransferase, U/L	29.56 ± 5.59	24.65 ± 4.54	21.10 ± 6.81	18.12 ± 3.90	18.33 ± 10.32	30.14 ± 6.47	25.52 ± 7.43	23.13 ± 7.14	21.50 ± 21.56	22.19 ± 33.48
Serum calcium, mmol/L	2.52 ± 0.08	2.50 ± 0.07	2.48 ± 0.07	2.48 ± 0.08	2.46 ± 0.08	2.51 ± 0.09	2.48 ± 0.07	2.49 ± 0.07	2.49 ± 0.08	2.49 ± 0.08
Albumin-corrected calcium, mmol/L	2.36 ± 0.07	2.32 ± 0.06	2.29 ± 0.07	2.27 ± 0.07	2.26 ± 0.07	2.36 ± 0.07	2.31 ± 0.06	2.30 ± 0.06	2.27 ± 0.07	2.26 ± 0.07
Inorganic phosphate, mmol/L	1.68 ± 0.13	1.60 ± 0.14	1.57 ± 0.18	1.36 ± 0.17	1.29 ± 0.14	1.68 ± 0.14	1.60 ± 0.15	1.60 ± 0.16	1.49 ± 0.24	1.26 ± 0.19
Alkaline phosphatase, mmol/L	230.40 ± 61.54	245.27 ± 62.34	262.76 ± 79.51	137.97 ± 64.02	78.21 ± 24.44	225.24 ± 61.24	238.37 ± 56.21	287.74 ± 85.97	252.91 ± 110.43	117.21 ± 48.65
Creatinine clearance, ml/min/1.73 m^2^	155.07 ± 21.78	149.78 ± 19.57	155.50 ± 22.61	138.44 ± 21.42	130.03 ± 17.05	154.21 ± 21.61	147.65 ± 22.47	143.37 ± 18.87	158.40 ± 26.86	136.70 ± 19.38
Triglyceride, mmol/L	0.80 ± 0.31	0.81 ± 0.32	1.00 ± 0.42	1.02 ± 0.43	0.95 ± 0.40	0.74 ± 0.30	0.77 ± 0.34	0.90 ± 0.42	0.95 ± 0.43	0.91 ± 0.39
Total cholesterol, mmol/L	4.03 ± 0.72	3.98 ± 0.68	3.88 ± 0.64	3.86 ± 0.68	3.74 ± 0.66	3.95 ± 0.70	3.97 ± 0.69	3.89 ± 0.65	3.58 ± 0.63	3.53 ± 0.60
Low density lipoprotein cholesterin, mmol/L	2.48 ± 0.65	2.31 ± 0.61	2.20 ± 0.58	2.17 ± 0.60	2.11 ± 0.57	2.37 ± 0.63	2.27 ± 0.62	2.24 ± 0.62	1.97 ± 0.57	2.04 ± 0.52
High density lipoprotein cholesterin, mmol/L	1.48 ± 0.32	1.58 ± 0.33	1.52 ± 0.31	1.49 ± 0.30	1.51 ± 0.31	1.53 ± 0.33	1.64 ± 0.35	1.55 ± 0.34	1.40 ± 0.31	1.34 ± 0.29
Blood glucose, mmol/L	4.49 ± 0.50	4.63 ± 0.50	4.69 ± 0.46	4.75 ± 0.48	4.75 ± 0.72	4.61 ± 0.49	4.75 ± 0.52	4.76 ± 0.47	4.82 ± 0.52	4.65 ± 0.55
Dyslipidemia	207 (17.78)	142 (9.74)	148 (14.22)	145 (15.93)	97 (11.36)	167 (12.74)	159 (10.28)	136 (12.58)	121 (15.36)	85 (16.19)
High TG, *n*(%)	21 (1.80)	25 (1.71)	69 (6.63)	73 (8.02)	38 (4.45)	17 (1.30)	26 (1.68)	56 (5.18)	47 (5.96)	22 (4.19)
High TC, *n*(%)	70 (6.01)	64 (4.39)	39 (3.75)	35 (3.85)	27 (3.16)	54 (4.12)	81 (5.24)	34 (3.15)	10 (1.27)	4 (0.76)
High LDL-C, *n*(%)	101 (8.68)	68 (4.66)	31 (2.98)	32 (3.52)	30 (3.51)	77 (5.87)	75 (4.85)	45 (4.16)	7 (0.89)	9 (1.71)
Low HDL-C, *n*(%)	73 (6.27)	44 (3.02)	49 (4.71)	37 (4.07)	29 (3.40)	62 (4.73)	38 (2.46)	40 (3.70)	76 (9.64)	52 (9.90)

*TG, triglyceride; TC, total cholesterol; LDL-C, low density lipoprotein-cholesterol; HDL-C, high density lipoprotein-cholesterol*.

Table 2 presents the differences between healthy children and those with dyslipidemia. The differences in albumin, creatinine, creatinine clearance, and blood glucose were non-significant. The serum calcium and albumin-corrected calcium level in those with dyslipidemia were higher than those in healthy children.

**Table 2:** Comparisons between normality and dyslipidemia.

	**Dyslipidemia**	**Non-dyslipidemia**	***t*/χ^2^**	***P***
*N*	1,407	9,272	−	−
**Sex**				
Female, *n*(%)	739 (13.62)	4,688 (86.38)	1.882	0.170
Male, *n*(%)	668 (12.72)	4,584 (87.28)		
Age, year	10.00 ± 4.59	10.06 ± 4.87	−0.380	0.706
**Age group**				
2–5 years, *n*(%)	374 (15.11)	2,101 (84.89)	43.602	<0.001
6–9 years, *n*(%)	301 (10.02)	2,704 (89.98)		
10–12 years, *n*(%)	284 (13.38)	1,838 (86.62)		
13–15 years, *n*(%)	266 (15.67)	1,432 (84.33)		
16–18 years, *n*(%)	182 (13.20)	1,197 (86.80)		
Height, cm	137.4 ± 26.86	136.9 ± 24.33	−0.640	0.520
Weight, kg	39.72 ± 21.20	36.02 ± 17.27	−6.230	<0.001
BMI, kg/m^2^	17.92 ± 3.52	19.20 ± 4.45	−10.250	<0.001
BMI z-score	0.49 ± 1.36	0.12. ± 1.21	−9.610	<0.001
Total Protein, g/L	74.63 ± 5.16	74.00 ± 4.76	−4.300	<0.001
Albumin, g/L	49.07 ± 2.80	49.15 ± 2.62	0.940	0.348
Creatinine, μmol/L	48.93 ± 15.95	48.09 ± 14.50	−1.870	0.062
Alanine aminotransferase, U/L	17.34 ± 19.44	13.78 ± 7.66	−6.790	<0.001
Aspartate aminotransferase, U/L	25.43 ± 22.70	23.86 ± 9.32	−2.550	0.011
Serum calcium, mmol/L	2.50 ± 0.08	2.49 ± 0.08	−4.400	<0.001
Albumin-corrected calcium, mmol/L	2.32 ± 0.08	2.31 ± 0.07	−5.430	<0.001
Inorganic phosphate, mmol/L	1.53 ± 0.23	1.55 ± 0.21	3.760	<0.001
Alkaline phosphatase, mmol/L	212.0 ± 91.89	218.9 ± 90.36	2.640	0.008
Creatinine clearance, mL/min/1.73 m^2^	148.0 ± 22.45	147.8 ± 25.00	0.250	0.801
Triglyceride, mmol/L	1.26 ± 0.63	0.81 ± 0.28	−26.930	<0.001
Total cholesterol, mmol/L	4.37 ± 1.12	3.81 ± 0.56	−18.450	<0.001
Low density lipoprotein cholesterin, mmol/L	2.79 ± 0.95	2.17 ± 0.50	−24.110	<0.001
High density lipoprotein cholesterin, mmol/L	1.31 ± 0.44	1.56 ± 0.30	20.230	<0.001
Blood glucose, mmol/L	4.71 ± 0.67	4.68 ± 0.50	−1.530	0.125

Based on the distribution of the serum calcium and albumin-corrected calcium, three categories were divided. The 25th and 75th percentile were 2.44 and 2.54 mmol/L for serum calcium levels and 2.25 and 2.54 mmol/L for albumin-corrected calcium levels. An increasing tendency in the rate of dyslipidemia both in serum calcium level and albumin-corrected calcium was observed, which was only statistically significant for albumin-corrected calcium (*p* for trend < 0.001). Except for high TG, a significant monotonic trend was revealed between dyslipidemia components and calcium levels (all *p* for trend < 0.001). The rate of high TC and high LDL-C increased with calcium levels, while low HDL-C decreases (Table 3). Similarly, the monotonic trends were found in the multivariable analysis. In the age-, sex-, and BMI z-score-adjusted model (Model 1), the highest categories of serum calcium and albumin-corrected calcium levels were both associated with higher probabilities for dyslipidemia, using the middle category as the reference. This association was not altered after further adjustment for several other covariates and confounding factors including albumin, alanine aminotransferase, creatinine, inorganic phosphate, alkaline phosphatase, and total protein. With the middle category of serum calcium level as the reference, the multivariable-adjusted ORs and 95% CIs of the lowest and the highest categories were 0.96 (0.82–1.12) and 1.29 (1.12–1.48), respectively, for total serum calcium levels and 1.06 (0.91–1.23) and 1.39 (1.21–1.60) for albumin-corrected calcium levels (Table 4). The standardized coefficients of TC, TG, LDL-C, and HDL-C for albumin-corrected calcium levels were 0.411, 0.031, −0.270, and −0.045 (all *p*-values < 0.05) in the linear regression model (Supplementary Table 2).

**Table 3:** The rate of dyslipidemia and components trends with serum calcium and albumin corrected calcium.

	**Serum calcium**						**Albumin-corrected calcium**					
	**T1**	**T2**	**T3**	**X^**2**^**	**P**	**P for trends**	**T1**	**T2**	**T3**	**X^**2**^**	**P**	**P for trends**
Cutoff values (less)	<2.44		≥2.54	-	-	-	<2.256		≥2.54	-	-	-
N	2,681	5,209	3,016	-	-	-	3,216	5,530	2,588	-	-	-
Dyslipidemia, *n*(%)	319 (11.90)	651 (12.50)	471 (15.62)	21.561	<0.001	0.742	337 (12.09)	679 (12.22)	425 (16.60)	33.526	<0.001	<0.001
High TG, *n*(%)	67 (2.50)	171 (3.28)	166 (5.50)	40.896	<0.001	0.677	97 (3.48)	195 (3.51)	112 (4.38)	4.222	0.121	0.090
High TC, *n*(%)	41 (1.53)	193 (3.71)	194 (6.43)	91.768	<0.001	<0.001	48 (1.72)	201 (3.62.)	179 (6.99)	78.923	<0.001	<0.001
High LDL-C, *n*(%)	60 (2.24)	223 (4.28)	203 (6.73)	57.926	<0.001	<0.001	66 (2.37)	219 (3.94)	201 (7.85)	101.365	<0.001	<0.001
Low HDL-C, *n*(%)	189 (7.05)	226 (4.34)	102 (3.38)	45.845	<0.001	<0.001	168 (6.03)	254 (4.57)	95 (3.71)	29.203	<0.001	<0.001
High Glucose, *n*(%)	80 (2.98)	149 (2.86)	133 (4.41)	15.534	<0.001	0.002	94 (3.37)	170 (3.06)	98 (3.83)	3.266	0.195	0.377

*Calcium categories were < 2.44, 2.44–2.53, and ≥ 2.54 mmol/L; Albumin corrected calcium categories were < 2.26, 2.26–2.53, and ≥ 2.54 mmol/L*.

*TG, triglyceride; TC, total cholesterol; LDL-C, low density lipoprotein-cholesterol; HDL-C, high density lipoprotein-cholesterol*.

**Table 4:** Multivariable-adjusted odds ratios and 95% confidence intervals of serum calcium and albumin corrected calcium associated with dyslipidemia.

		**Serum calcium**			**Albumin-corrected calcium**		
		**T1**	**T2**	**T3**	**T1**	**T2**	**T3**
Crude Model	Dyslipidemia	0.946 (0.820–1.091)	Ref	1.296 (1.140–1.473)	0.988 (0.859–1.136)	Ref	1.431 (1.254–1.632)
	High TG	0.755 (0.567–1.006)	Ref	1.716 (1.379–2.135)	0.991 (0.774–1.270)	Ref	1.258 (0.993–1.595)
	High TC	0.404 (0.287–0.567)	Ref	1.787 (1.456–2.192)	0.467 (0.340–0.642)	Ref	2.004 (1.629–2.465)
	High LDL-C	0.512 (0.383–0.683)	Ref	1.614 (1.327–1.962)	0.591 (0.447–0.781)	Ref	2.077 (1.705–2.531)
	Low HDL-C	1.672 (1.370–2.041)	Ref	0.772 (0.608–0.979)	1.339 (1.096–1.636)	Ref	0.805 (0.633–1.024)
	High Gluc	1.045 (0.793–1.376)	Ref	1.567 (1.235–1.988)	1.106 (0.856–1.429)	Ref	1.262 (0.980–1.625)
Adjusted Model 1	Dyslipidemia	0.978 (0.844–1.134)	Ref	1.271 (1.115–1.449)	0.945 (0.814–1.096)	Ref	1.487 (1.293–1.711)
	High TG	0.706 (0.524–0.950)	Ref	1.800 (1.436–2.256)	0.734 (0.565–0.954)	Ref	1.828 (1.416–2.361)
	High TC	0.421 (0.296–0.600)	Ref	1.702 (1.379–2.100)	0.467 (0.331–0.659)	Ref	1.865 (1.492–2.331)
	High LDL-C	0.561 (0.416–0.757)	Ref	1.474 (1.205–1.803)	0.653 (0.482–0.885)	Ref	1.732 (1.402–2.139)
	Low HDL-C	1.736 (1.413–2.133)	Ref	0.734 (0.575–0.936)	1.337 (1.078–1.657)	Ref	0.811 (0.630–1.042)
	High Gluc	0.934 (0.702–1.242)	Ref	1.636 (1.285–2.084)	0.854 (0.651–1.119)	Ref	1.727 (1.320–2.258)
Adjusted Model 2	Dyslipidemia	0.927 (0.794–1.083)	Ref	1.359 (1.181–1.564)	1.006 (0.866–1.169)	Ref	1.449 (1.258–1.669)
	High TG	0.768 (0.563–1.047)	Ref	1.685 (1.319–2.153)	0.758 (0.582–0.988)	Ref	1.829 (1.412–2.370)
	High TC	0.440 (0.307–0.632)	Ref	1.629 (1.300–2.042)	0.460 (0.326–0.650)	Ref	1.875 (1.498–2.347)
	High LDL-C	0.556 (0.408–0.757)	Ref	1.490 (1.201–1.848)	0.657 (0.485–0.891)	Ref	1.723 (1.394–2.129)
	Low HDL-C	1.520 (1.220–1.893)	Ref	0.863 (0.668–1.115)	1.532 (1.231–1.907)	Ref	0.763 (0.592–0.983)
	High Gluc	0.891 (0.662–1.200)	Ref	1.711 (1.322–2.215)	0.830 (0.632–1.089)	Ref	1.759 (1.344–2.303)
Adjusted Model 3	Dyslipidemia	0.956 (0.817–1.118)	Ref	1.288 (1.117–1.484)	1.056 (0.907–1.231)	Ref	1.390 (1.206–1.603)
	High TG	0.818 (0.598–1.119)	Ref	1.554 (1.212–1.994)	0.832 (0.636–1.088)	Ref	1.685 (1.297–2.189)
	High TC	0.464 (0.322–0.667)	Ref	1.513 (1.203–1.901)	0.494 (0.349–0.700)	Ref	1.765 (1.407–2.214)
	High LDL-C	0.573 (0.420–0.781)	Ref	1.398 (1.124–1.739)	0.688 (0.505–0.936)	Ref	1.637 (1.322–2.028)
	Low HDL-C	1.547 (1.237–1.933)	Ref	0.822 (0.635–1.065)	1.585 (1.269–1.980)	Ref	0.739 (0.573–0.954)
	High Gluc	0.875 (0.648–1.182)	Ref	1.696 (1.307–2.203)	0.824 (0.625–1.086)	Ref	1.763 (1.343–2.314)

*Model 1 adjusted for age, sex, and BMI z-score (continuous)*.

*Model 2 further adjusted for albumin (continuous), alanine aminotransferase (continuous), and creatinine (continuous)*.

*Model 3 further adjusted for inorganic phosphate (continuous), alkaline phosphatase (continuous), and total protein (continuous)*.

*Calcium categories were < 2.44, 2.44–2.53, and ≥ 2.54 mmol/L; Albumin corrected calcium categories were < 2.26, 2.26–2.53, and ≥ 2.54 mmol/L*.

*TG, triglyceride; TC, total cholesterol; LDL-C, low density lipoprotein-cholesterol; HDL-C, high density lipoprotein-cholesterol; Ref, reference*.

**Figure 2:**
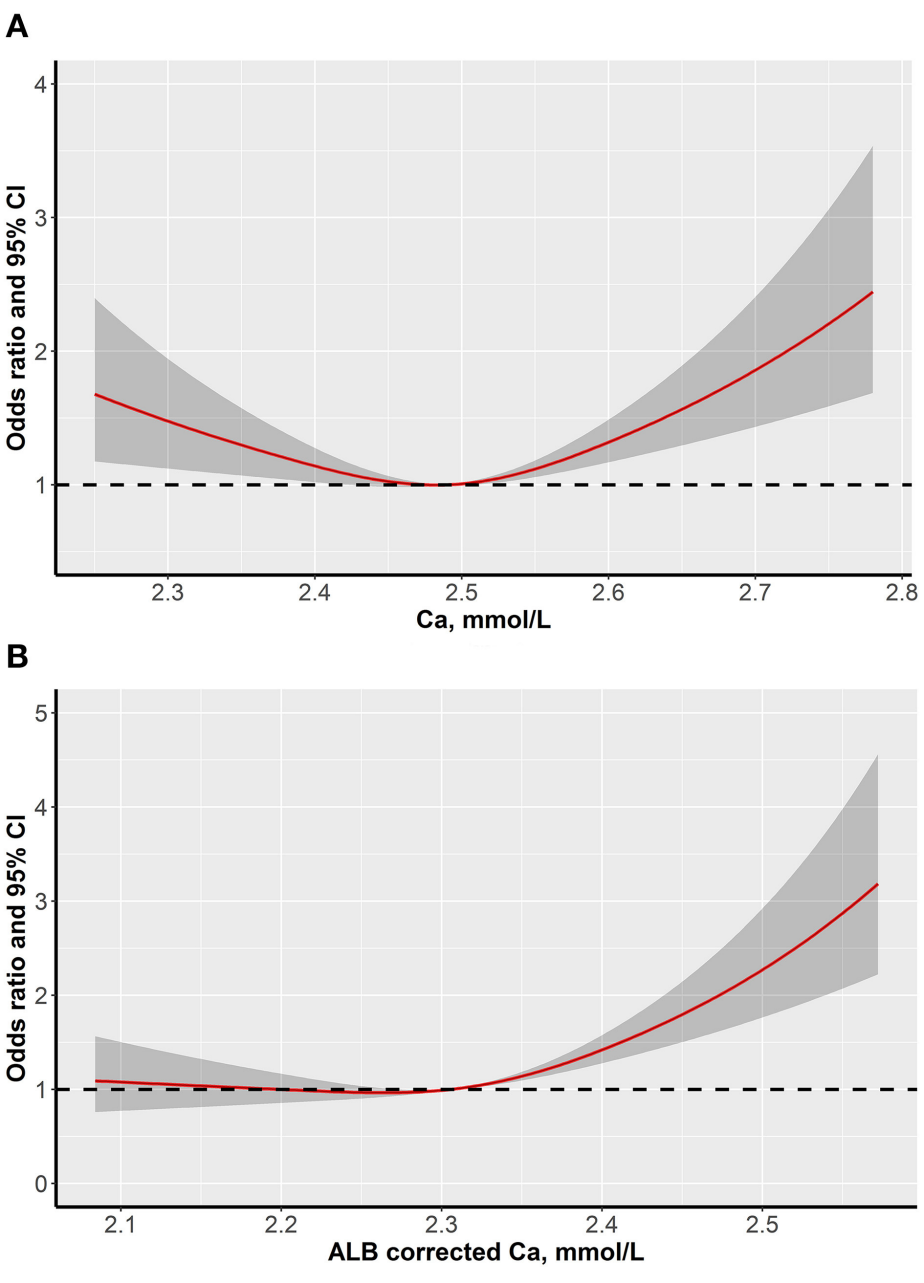
Dose-response associations between serum calcium levels and dyslipidemia (**A** is serum calcium levels and **B** is albumin-corrected calcium levels).

The restricted cubic spline model indicated an L-shaped dose-response relation between serum calcium levels and the probability of dyslipidemia after adjustment for multiple potential confounding factors (*p* for nonlinear < 0.001) (Figure 2A). However, the risk of dyslipidemia only showed a monotonically increasing tendency when the albumin-corrected calcium level was beyond 2.50 mmol/L after adjustment of confounding factors as well (*p* for nonlinear < 0.001) (Figure 2B).

## Discussion

The physiological function of calcium is to not only participate in osteogenesis and function within osteoclasts but is also involved in neurotransmitter release, hormone secretion, maintenance of blood coagulation, and homeostasis (12). In plasma, about 50% of calcium is free or ionized, 40% is bound to plasma proteins, and 10% is complexed with anions such as bicarbonate, phosphate, lactate, and citrate (4). Albumin is the predominant calcium-binding protein with 1 g/L of albumin binding to approximately 0.8 mg/dl of calcium (24).

Epidemiological studies found that higher serum calcium levels and albumin-corrected calcium levels were associated with an increased risk of developing metabolic syndrome, diabetes, and hypertension (6, 27–29).

The US National Cholesterol Education Program (NCEP) proposed a set of cut-off values for lipid concentrations to define dyslipidemia aged 2–19 years. The definition of dyslipidemia based on Chinese expert consensus in this study has a little bit difference from the NCEP pediatric cutpoints, which was the same as in high TC (≥5.18 mmol/L) and high LDL-C (≥3.37 mmol/L), but higher in low HDL-C ( ≤ 1.04 mmol/L in China vs. ≤ 0.91 mmol/L in US) and high TG (≥1.70 mmol/L in China vs. ≥1.13 mmol/L for 2–9 years and ≥1.47 mmol/L for 10–19 years in the USA) (22, 23).

The results from Tables 3, 4 show that among several indicators of dyslipidemia, there was no statistical significance in high TG rates among percentiles of calcium levels, whether using the total serum calcium level or albumin-corrected levels.

A similar trend was also confirmed in multivariable models. All other biomarkers of dyslipidemia separately depicted linear increasing trends. This difference in cholesterol and triglyceride associated with calcium could be caused by the distinctions of metabolism and regulation of triglyceride and cholesterol. The former is regulated by hormones, such as insulin, while the latter is regulated by lipoproteins.

In a recent epidemiological investigation of osteoporosis prevention in postmenopausal women, long-term calcium supplementation could affect the serum cholesterol level of a female population. Another research revealed high calcium intake increases in rat serum levels of TC, TG, LDL-C, and HDL-C and eventually leads to calcium depositions in the carotid artery (30). The association between calcium intakes and lipid metabolism had been found in both animal researches and population studies. The mechanism was thought to be that long-term calcium supplementation converted the smooth muscle cells into osteoblast-like cells, which caused the formation of matrix vesicles, which secrete mineralized crystals, leading to smooth muscle cell apoptosis (31).

Studies showed that fluctuating calcium concentrations could reflect not only the exogenous intake but also the endogenous capability of maintaining homeostasis (32), which was tightly regulated by multiple negative feedback loops involving several target organs and hormones (33). Another study observed a significant correlation between serum calcium levels and TC, HDL-C, LDL-C, non-HDL-C, and TG both in postmenopausal women and in men (34). The potential mechanisms involved in this were hypothesized but remain unclear. Firstly, higher serum calcium levels resulted in smaller concentrations of serum pyrophosphates and greater tissue calcification (6). A study explored the effect of blood lipid markers on nephrolithiasis in children; it suggested that children with calcium oxalate stones accompanied with the existence of abnormal lipid metabolism (35).

Secondly, mitochondrial and free calcium dynamics formed a homeostatic circuit that compartmentalized and coordinated energy metabolism, ROS production, signal transduction, proliferation, and cell death due to internal and environmental cues (2, 36, 37). It was confirmed that serum calcium levels were directly correlated to glucose intolerance, insulin resistance, impaired glucose metabolism, early-phase insulin secretion, and diabetes (12). All of the aforementioned factors went hand in hand with dyslipidemia. Another explanation indicated the influence of hepatic catabolism, especially in estrogen-deficient conditions (38). Presence of calcium could decrease cholesterol catabolism, and stimulates lipids synthesis. In more details, higher calcium levels could decrease the activity of the 7a-hydroxylase (CYP7A), an enzyme involved in the cholesterol catabolism, and stimulates sterol regulatory element-binding protein (SREBP)-1c expression, which is a transcription factor involved in *de novo* lipid synthesis (39).

In addition, the regulation of calcitonin, vitamin D, and the parathyroid hormone might affect lipid metabolism as well. All hypotheses mentioned above would be explored and tested in further experiments.

Our study results suggested that serum calcium or albumin-corrected calcium levels above 2.50 mmol/L in children might seem to be associated with the risk of dyslipidemia, although they failed to meet the clinical diagnosis cutoff value for hypercalcemia. Under the condition of the uncertain association, arbitrarily overuse of calcium, VD supplements, and the coexistence of excessive nutrition and lack of exercise among children on top of the epidemic period, all these would increase the risk of cardiovascular disease in children. Further clinical and basic researches are needed to clarify the mechanisms involved. If such association does exist and the mechanism should be clarified, it might lead to new targets for the clinical treatment of dyslipidemia, as well as to more rational and scientific considerations for the use of calcium supplements in children from a public health perspective.

Our study depicted an interesting phenomenon that there might be a potential association between serum calcium levels and dyslipidemia in a large and nationally representative sample of Chinese children. Nonetheless, this study had several limitations. Firstly, the cross-sectional nature of the PRINCE study made it difficult to determine a causal relationship between serum calcium level and dyslipidemia. The primary objective of the PRINCE study was to establish the pediatric reference intervals involved. Thus, the association between serum calcium level and dyslipidemia was still not clear whether it would exist in the natural population from community or in the case-control study, which would be also the direction for further studies to confirm it.

Secondly, due to the lack of physical activity data, dietary investigation including calcium intakes or supplement, and other relevant hormones or biomarkers (e.g., vitamin D, calcitonin, and parathyroid hormone), we cannot further analyze their effects on both, serum calcium levels and lipid metabolism. Further epidemiological or clinical studies that would take into account the corresponding factors should be conducted for validation purposes in the future.

Lastly, the continuous age-dependent trends in children should have been considered; the standardized deviation score (SDS) methods, which would adjust the influence by age and sex, could be applied when exploring the association between calcium levels and dyslipidemia. Further research in community child populations that focus on calcium SDS and other potential confounding factors should be designed and carried out.

## Conclusion

We found that individuals with higher levels of serum calcium were associated with increased risk of dyslipidemia in a healthy sample of Chinese children. It is well-established that calcium deficiency is linked to some diseases and should be treated with calcium fortification or supplementation such as vitamin D supplements. However, potential adverse health outcomes, such as change in lipid metabolism and vascular calcification can be associated with high calcium volumes resulting from excessive supplementation and need to be explored in future studies.

## Data Availability Statement

The raw data supporting the conclusions of this article will be made available by the authors, without undue reservation.

## Author Contributions

XP and WS conceived the present study idea. YP, LH, XN, SC, RY, YL, and YC conducted the study. YP, XN, SC, and RY conducted the data collection and data manage. LH and YC performed quality control of the sample collection and laboratory tests. YP and RY performed the data analysis. YP drafted the manuscript. All authors reviewed and approved the final manuscript and took responsibility for the reliability and freedom from bias of the data presented and their interpretation.

## Conflict of Interest

The authors declare that the research was conducted in the absence of any commercial or financial relationships that could be construed as a potential conflict of interest.

## Publisher's Note

All claims expressed in this article are solely those of the authors and do not necessarily represent those of their affiliated organizations, or those of the publisher, the editors and the reviewers. Any product that may be evaluated in this article, or claim that may be made by its manufacturer, is not guaranteed or endorsed by the publisher.
