# Supplementary material for: The Role of Serum Calcium Levels in Pediatric Dyslipidemia: Are There Any?
Source: Front Pediatr. 2021 Aug 9;9:712160. doi: 10.3389/fped.2021.712160 (PMC8380842; doi:10.3389/fped.2021.712160)
Supplement: Supplementary file 1 [file Table_1.docx]

**Supplementary Table 1** The detection methods of biochemical analytes

| Items | Abbreviation | Approaches on Cobus C702 | Measurement unit |
| --- | --- | --- | --- |
| Total protein | TP | Colorimetry (Biuret) | g/L |
| Albumin | ALB | Colorimetry (Bromocresol green) | g/L |
| Creatinine | Cr | Enzymatic methods | μmol/L |
| Total cholesterol | TC | Colorimetry (Enzymatic methods) | mmol/L |
| Glucose | GLU | Colorimetry (Hexokinase method) | mmol/L |
| Serum calcium | Ca | Colorimetry (Arsenazo III) | mmol/L |
| Inorganic phosphate | P | Ultraviolet spectrophotometry (Molybdate) | mmol/L |
| Alkaline phosphatase | ALP | Colorimetry (Para-nitrophenyl phosphate) | U/L |
| Alanine aminotransferase | ALT | Kinetic rate method (Without pyridoxal-5'-phosphate) | U/L |
| Triglyceride | TG | Colorimetry (Enzymatic methods) | mmol/L |
| High density lipoprotein-cholesterol | HDL-C | Colorimetry (Homogeneous Enzymatic methods) | mmol/L |
| Low density lipoprotein-cholesterol | LDL-C | Colorimetry (Homogeneous Enzymatic methods) | mmol/L |
