# Supplementary material for: The Role of Serum Calcium Levels in Pediatric Dyslipidemia: Are There Any?
Source: Front Pediatr. 2021 Aug 9;9:712160. doi: 10.3389/fped.2021.712160 (PMC8380842; doi:10.3389/fped.2021.712160)
Supplement: Supplementary file 2 [file Table_2.docx]

**Supplementary Table 2** The regression analysis between albumin-corrected calcium and serum lipid

| Variables | Beta coefficient | Standardized coefficient | P |
| --- | --- | --- | --- |
| Age, year | -0.007 | -0.405 | <0.001 |
| Height, cm | -0.002 | -0.720 | <0.001 |
| Weight, kg | 0.003 | 0.646 | <0.001 |
| BMI, kg/m^2^ | -0.004 | -0.219 | <0.001 |
| Total Protein, g/L | 0.005 | 0.296 | <0.001 |
| Albumin, g/L | -0.007 | -0.266 | <0.001 |
| Creatinine, μmol/L | 0.001 | 0.198 | <0.001 |
| Inorganic phosphate, mmol/L | 0.024 | 0.067 | <0.001 |
| Alkaline phosphatase, mmol/L | 0.000 | 0.048 | <0.001 |
| Creatinine clearance, mL/min/1.73m^2^ | 0.000 | 0.041 | 0.004 |
| Triglyceride, mmol/L | 0.006 | 0.031 | 0.017 |
| Total cholesterol, mmol/L | 0.045 | 0.411 | <0.001 |
| Low density lipoprotein cholesterin, mmol/L | -0.032 | -0.270 | <0.001 |
| High density lipoprotein cholesterin, mmol/L | -0.010 | -0.045 | 0.037 |
| Blood glucose, mmol/L | 0.016 | 0.110 | <0.001 |
